# Soilless culture as a novel strategy for maximizing growth performance and fiber yield in industrial hemp (*Cannabis sativa* L.)

**DOI:** 10.1186/s12870-026-08423-y

**Published:** 2026-03-04

**Authors:** Mehmet Yusuf Orcan, Filiz Akbaş

**Affiliations:** 1 Food Analyses Application and Research Center, Batman University, Batman, Türkiye; 2 Depertmant of Biology, Faculty of Science and Letters, Batman University, Batman, Türkiye

**Keywords:** *Cannabis sativa* L., Agricultural biotechnology, Bast fiber crops, Controlled environment cultivation, Deep water culture, Silicon

## Abstract

**Background:**

Hemp (*Cannabis sativa* L.) is one of the oldest cultivated plants in human history. It is a valuable plant that has economic value for all industrial branches. The hemp plant has fibers of the highest quality. Accordingly, research aiming to enhance the fiber yield of hemp has significant agronomic and industrial relevance.

**Methods:**

To this end, three soilless media treatments were applied to the modified Hoagland solution. The effects of N: P:K/255:45:267 (A1), A1 + 10 mg/L silicon (A2), and A1 + 1 mg/L indole butyric acid (A3) on plant growth and fiber yield were investigated in Narlı hemp (*Cannabis sativa* L.), a registered local variety (Türkiye), in deep water culture and soil media (A4).

**Results:**

The analysis determined that the soilless medium was more productive than the soil medium in terms of root fresh-dry weight, stem fresh-dry weight, stem length, stem thickness, leaf length and width, relative water content (RWC), photosynthetic pigment content, fiber content, and total cellulose content. According to these results, the lowest values were obtained in the soilless medium in terms of malondialdehyde (MDA) and leaf secondary metabolite (cannabidiol: CBD, tetrahydrocannabinol: THC, and cannabinol: CBN) contents, which indicate stress in the plant. CBD was measured %0.0213, %0.0134, %0.0395, %0.0326, THC was measured %0.002, %0.0017, %0.0032, %0.0033, CBN was measured %0.0003, %0.0002, %0.0002, %0.0009 (A1, A2, A3, A4 respectively). Considering all the parameters studied, the hemp plant grown in the deep water culture medium containing Si yielded the best results among the treatments. The fiber content was 29.12% in A2, 24.02% in A1, 19.99% in A4, and 13.74% in A3. The cellulose content was determined to range from 61% to 82% on average in the treatments tested. The highest total cellulose content of 82% was obtained from treatment A2.

**Conclusion:**

The current study revealed that silicon (Si) treatment significantly increased fiber and cellulose contents. Incorporating such treatments into production models is regarded as a cost-effective and efficient strategy.

## Introduction

Hemp (*Cannabis sativa* L.) is one of the oldest cultivated plants in human history, with origins dating back to 8000 BC. It was used as a medicinal plant in China as early as 2700 BC. The hemp plant is believed to have been introduced from Asia to Europe during this period, and its cultivation continues worldwide today [1]. The hemp plant was first named *Cannabis sativa* L. by Carlous Linnaeus in 1753. In 1785, the French biologist Jean-Baptiste Lamarck described the species *Cannabis indica*, which morphologically differs from *C. sativa* and originates from India. In 1924, Janischevsky discovered *Cannabis ruderalis* in Russia, which has different characteristics from the other two species. Although some researchers consider *C. indica* and *C. ruderalis* to be subspecies of *C. sativa*, it can be said that the three species of cannabis have been systematically defined according to the common opinion [2, 3].

Hemp is an annual, short-day, herbaceous, flowering plant belonging to the *Cannabinaceae* family [4]. Hemp is a valuable plant, all parts of which can be utilized. Moreover, it has economic value for all industrial branches and superiorities in almost all areas of use. For example, it has been determined that the fiber and mineral mixture is more advantageous than concrete in the production of roof and wall materials, considering weight, durability, and quality criteria. Additionally, the rate of humidity decreases and heat insulation is at the best level in constructions where this mixture made with hemp is used [3]. Another important advantage of the hemp plant is that all these structures are suitable for recycling and do not cause environmental pollution [5].

Industrial hemp fibers are preferred in many areas due to high strength, abrasion resistance, breathability, high moisture absorption, non-pilling, anti-bacterial properties, UV protection, and high electrostatic properties. According to various sources, the hemp plant is used in over 50,000 products [6]. Biodegradable hemp fiber has been preferred in the garment industry in recent years due to its properties, such as antimicrobial and moisture absorbency, as well as its industrial uses. Along with the textile sector, hemp fibers are also widely used in the production of composite materials [7]. Due to its strong bonding with glue and other polymer materials, hemp is commonly used in areas such as door and seat panels in the automobile industry. At the same time, it is especially preferred for upholstery fabrics in the furniture industry. Hemp fibers stand out among other fibers in terms of production costs. Thus, when composite insulations made of hemp were used to replace composite insulations made of glass fiber, 23% lighter and 20% cost savings were achieved [8].

However, cannabis cultivators still lack scientific information about optimal growing conditions, such as the supply of mineral nutrients, to help maximise crop yields, quality, and profits while minimising environmental impacts [9]. The use of various cultivation methods in hemp cultivation has been studied to increase the plant yield and quality elements, and hemp is cultivated more widely using alternative agricultural techniques, such as in vitro micropropagation, indoor cultivation, and soilless farming. All these considerations indicate that the present study aims to evaluate the potential of hydroponic cultivation of industrial hemp to shorten the harvest period and enhance yield performance. The effects of N, P, K nutrients, silicon, and indol butyric acid (IBA) on plant growth and fiber yield in soilless media has been investigated in the current study.

## Materials and methods

Narlı [10] hemp cultivar seeds, registered as fiber-type hemp, were used as the starting material in the study. The seeds were obtained Republic of Türkiye Ministry of Agriculture and Forestry, Black Sea Agricultural Research Institute. Legal permissions to carry out the research were obtained from the Ministry of Agriculture and Forestry and the Governorship of Batman (Türkiye). The current study was conducted in a controlled environment, at 24–26 °C ambient temperature, 60–65% ambient humidity, 210 µmol.m^-2^.s^-1^ PPFD, and 16:8 h light/dark photoperiod. Plants were grown with the modified Hoagland nutrient solution [11] in deep water culture. Plants were also grown in pots with soil under the same conditions, and changes in hemp grown in soil and soilless media were investigated.

### Seed germination

In light of preliminary studies showed that the germination medium containing a mixture of unfertilised peat, soil, and perlite (1:1:1) performed the best. Hemp seeds were sown at a depth of 2–3 cm in small pots with soil, one seed in a pot, and left to germinate.

### The vegetation growth conditions of hemp plants

**Fig. 1:**
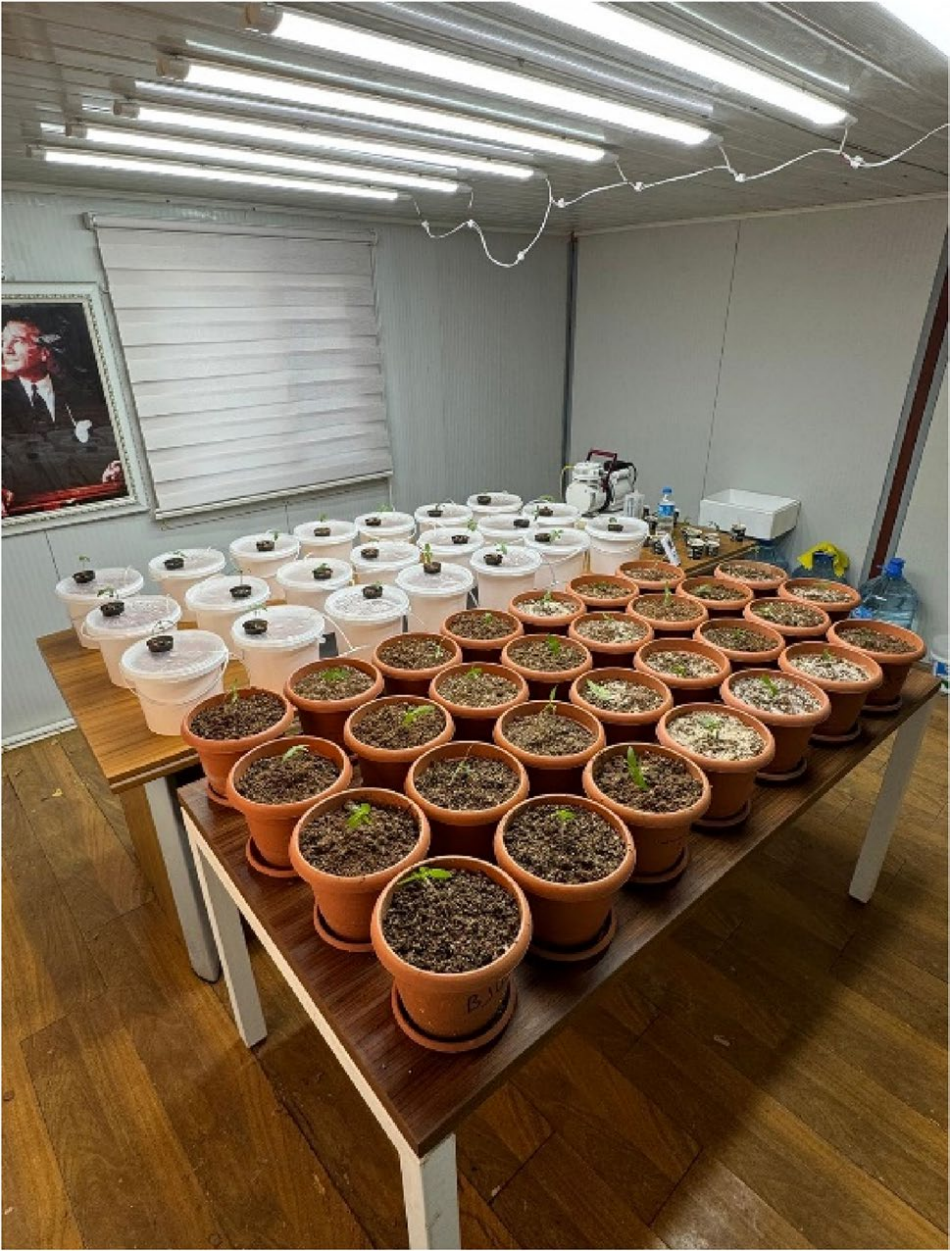
Plant growth room

After germination, plants that had developed for 2 weeks and produced the 2nd pair of true leaves were transferred to hydroponic pots with clay pebble support and to the deep water culture medium, with one plant in a separate container. Plants that produced the second pair of true leaves were transferred to soil pots containing peat, soil, and perlite in a 1:1:1 ratio. All the pots prepared were left to develop in the growth chamber under controlled environmental conditions (Fig. 1).

### Experimental design and nutrient treatments

A preliminary study was conducted to determine the optimal nutrient requirements of hemp plants in the deep water culture. In this study, three different nutrient media containing N, P, and K elements at the ratios of 50:10:50, 100:20:100, and 150:30:150 mg/L, respectively, were added to the half-strength Hoagland nutrient medium. The present study determined that the best soilless growing medium was 1/2 Hoagland + 150:30:150 N: P:K according to total plant biomass, stem thickness, and fiber yield parameters.

Based on the elements and quantities used, the 1/2 Hoagland solution provided approximately 105 mg/L nitrogen (N), 117 mg/L potassium (K), 15 mg/L phosphorus (P), 80 mg/L calcium (Ca), 64 mg/L sulfur (S), and 48 mg/L magnesium (Mg) (Table 1). When additional 150:30:150 mg/L of N: P:K was supplemented to the 1/2 Hoagland medium, the final nutrient concentrations reached 255 mg/L for N, 45 mg/L for P, and 267 mg/L for K. Silicon (Si) was prepared from Na_2_SiO_3_ (Sigma 307815), while IBA was directly obtained from a commercial form (Sigma - I5386 CAS: 133-32-4) by dilution.

**Table 1:** Content of modified Hoagland solution

Compound	Amount in stock solution (g/L)	Amount in nutrient (ml/L)
KNO_3_	101	3
Ca(NO_3_)_2_•4H_2_O	236	2
MgSO_4_•7H_2_O	493	1
NH_4_H_2_PO_4_	57.5	1
H_3_BO_3_	2.86	1
MnCl_2_	1.81	1
ZnSO_4_.7H_2_O	0.22	1
CuSO_4_.5H_2_O	0.08	1
Na_2_MoO_4_.2H_2_O	0.12	1
Fe EDDH	15	1.5

To determine plant growth and fiber yield in hemp plants, four different treatments were applied in the current study. In three treatments, plants were grown in the deep water culture medium, whereas they were grown in the soil medium in one treatment.

- A1: 1/2 Hoagland + 150:30:150 N:P:K
- A2: 1/2 Hoagland + 150:30:150 N:P:K + 10 mg/L Si
- A3: 1/2 Hoagland + 150:30:150 N:P:K + 1 mg/L IBA
- A4: Soil (peat:soil:perlite/1:1:1)

At the end of the development period, which lasted approximately 8 weeks (62 days), hemp plants were harvested. A series of analyses performed mentioned below, then evaluated the effects of the treatments on plant growth and fiber yield.

### Root fresh and dry weight (g)

Roots were separated from the plant and washed with tap water. After residual nutrients were removed, the roots were rinsed with distilled water and placed in an oven at 35 °C for 1–2 h to allow surface moisture to evaporate. The roots were then weighed. Afterward, to determine the root dry weight, the roots whose fresh weights had been measured were dried until their weights stabilized. Subsequently, root dry weights were recorded.

### Stem fresh and dry weight (g)

The leaves and side branches of the harvested plants were removed, and the stems were separated. After being kept at room temperature for 2 h to reduce surface moisture, the stems’ fresh weight was measured using an analytical balance, and the average fresh weight was calculated. The stems, whose fresh weights had been measured, were dried in an oven at 55 °C until a constant weight was achieved. The average stem dry weight was then determined using an analytical balance.

### Stem length (cm)

At the end of the growth period, the average stem length was calculated by measuring the distance from the base of the main stem (the point where the root ends and the aerial parts begin) to the apex.

### Stem thickness (g)

The stem diameter was measured for each sample at a point 40 cm above the ground, corresponding to the beginning of the plant’s green part, using a digital caliper. The average of 7 plants was used for the parameters above.

### Leaf length (cm)

The average leaf length was determined by measuring the distance from the tip to the base of each leaf using a digital caliper. Samples were collected from three randomly selected hemp plants.

### Relative water content (%RWC)

To determine %RWC, the fresh weights of three randomly selected hemp leaves were measured, and then the leaves were soaked in sterile distilled water for 6 h, following which their turgid weights were recorded. The samples were dried in an oven at 55 °C until a constant weight was achieved, and their dry weights were determined. %RWC was calculated as a percentage using the formula below [12].

$$\mathbf{Relative}\;\mathbf{water}\;\mathbf{content}\left(\%\mathbf{RWC}\right)\;=\;\left[\left(\mathbf{FW-\mathbf{DW}}\right)\;/\;\left(\mathbf{TW}-\mathbf{DW}\right)\right]/\times\boldsymbol{100}$$

FW=fresh weight, DW = dry weight, TW=turgid weight.

### Photosynthetic pigments (mg/g FW)

The content of photosynthetic pigments was measured in triplicate. Chlorophyll *a*, chlorophyll *b*, and carotenoid contents in hemp plants were measured according to the method described by [13]. For each treatment, 0.25 g of randomly selected fresh leaf samples were homogenized in a mortar with 2 mL of 80% acetone. The homogenate was filtered through filter paper, and the volume was adjusted to 5 mL with 80% acetone. The extracts were then centrifuged at 5000 rpm for 5 min, and absorbance values were recorded spectrophotometrically at 663 nm for chlorophyll *a*, 645 nm for chlorophyll *b*, and 480 nm for carotenoids.

### Malondialdehyde (MDA) content (µmol/g FW)

To assess cell membrane damage in leaf tissues, the content of MDA, a final product of lipid peroxidation, was determined using the thiobarbituric acid (TBA) assay, as described by [14]. To this end, leaf samples randomly collected from all cultivated plants were ground in liquid nitrogen. Then, 100 mg of the frozen leaf tissue was weighed, and 2 mL of 5% trichloroacetic acid (TCA) was added. The samples were homogenized using a mortar and pestle. The homogenate was centrifuged at 10,000 rpm for 15 min at 25 °C. Subsequently, 1 mL of the supernatant was mixed with 1 mL of 20% TCA containing 0.5% TBA. The mixture was incubated in a hot water bath at 95 °C for 1 h to allow the reaction to occur and then rapidly cooled in an ice bath to stop the reaction. Absorbance was measured at 532 nm using a spectrophotometer, with a blank containing 20% TCA and 0.5% TBA.

### Leaf secondary metabolites content (%w/w)

The contents of some secondary metabolites, cannabidiol (CBD), cannabinol (CBN), and delta9-tetrahydrocannabinol (Δ⁹-THC), in the plants were quantified to determine changes in these compounds during plant development. Leaf samples randomly collected from all treatment groups were dried at room temperature in the absence of light. From each of seven plants, 3 g of the dried leaf material were weighed and extracted with 20 mL of methanol using a shaker-incubator at 130 rpm for 24 h. The analyses were performed using the gas chromatography (GC) system (Thermo Trace 1300) coupled with the TSQ 8000 Evo mass spectrometer (MS), equipped with the AI/AS 3000 automatic sampler. A Restek Rxi-5ms capillary column (30 m × 0.25 mm × 0.25 μm, cat. #13423) was used in the study. Sample injection was performed in split mode (split ratio 1:10) using a 1 µL injection volume, with an inlet temperature of 280 °C. Helium was used as the carrier gas at a flow rate of 1 mL/min. The MS transfer line temperature was set to 300 °C, and the ion source temperature to 250 °C. The column oven temperature program was as follows: the initial temperature was set to 20 °C and held for 2 min, followed by a ramp of 10 °C/min to a final temperature of 320 °C, which was held for 5 min. The total run time was 19 min.

### Fiber content (%w/w)

After the side branches and leaves were removed, the harvested hemp plants were soaked in pure water at room temperature for 3–5 days to undergo the retting process. The separated stems and fibers were then dried at room temperature until a constant weight was achieved. The average fiber content was calculated based on the method described by [15], using fiber samples from seven hemp plants.

$$\mathbf{Fc}\left(\%\right)\;=\;\left(\mathbf{DW}_\mathbf{fibre}\;\times\;\boldsymbol{100}\right)\;/\;\mathbf{DW}_\mathbf{total}$$

Fc: fiber content, DW_fiber_: dried fibers weight, DW_total_: the total dried weight.

### Total cellulose content (%w/w)

Fibers obtained from the stems of seven plants were analyzed according to the method described by [16], and the rate of change in total cellulose content was calculated.

Statistical analyses were conducted using the SPSS software package (Standard Version 20.0 for Windows). Differences between the control and treatment groups were evaluated using one-way analysis of variance (ANOVA), and mean comparisons were conducted using Duncan’s multiple range test at a significance level of *p* ≤ 0.05 [17]. Results were presented mean ± standart devation (SD).

## Results and Discussion

The study used seeds of the local Narlı variety of industrial hemp (*Cannabis sativa* L.) as a material. Root and stem fresh and dry weights, stem length and thickness, leaf length and width, leaf relative water content, photosynthetic pigment content, MDA content, leaf secondary metabolite content, fiber content, and cellulose content were measured and analyzed to assess the physiological development, fiber yield, and potential stress factors caused by growing conditions in the plants harvested at the end of the 77-day development period after they were left to germinate. The results for these parameters are given below.

### Plant root fresh and dry weight

Table 2 contains data on the root fresh and dry weights of Narlı hemp plants grown in soil and deep water culture.

**Table 2:** Fresh and dry weights of roots of hemp plants with different treatments

Treatment	Root Fresh Weight (g)	Root Dry Weight(g)
A1	3.05±0.14^a^	0.52±0.02^ba^
A2	3.2±0.46^a^	0.54±0.03^a^
A3	1.27±0.03^b^	0.37±0.03^c^
A4	1.92±0.64^ba^	0.42±0.03^cb^

A1: 1/2 Hoagland + 150:30:150 N: P:K

A2: 1/2 Hoagland + 150:30:150 N: P:K + 10 mg/L Si

A3: 1/2 Hoagland + 150:30:150 N: P:K + 1 mg/L IBA

A4: Soil media (peat: soil: perlite / 1:1:1)

* Values in each column marked with different letters indicate statistically significant differences at *p* ≤ 0.05

* The mean was calculated from data obtained from 7 plants

An examination of root fresh weight between the treatment groups showed that there were general differences, especially pronounced in plants grown in deep water culture and soil medium (except A3 treated with IBA). According to the data in Table 2, plants in Si-treated A2 had the highest value (3.2 g) in comparison with the other treatments. In terms of root fresh weight, A2 was followed by A1 (3.05 g) and A4 (1.92 g), respectively. A3 was considered the treatment with the lowest root fresh weight (1.27 g) in the study.

**Fig. 2:**
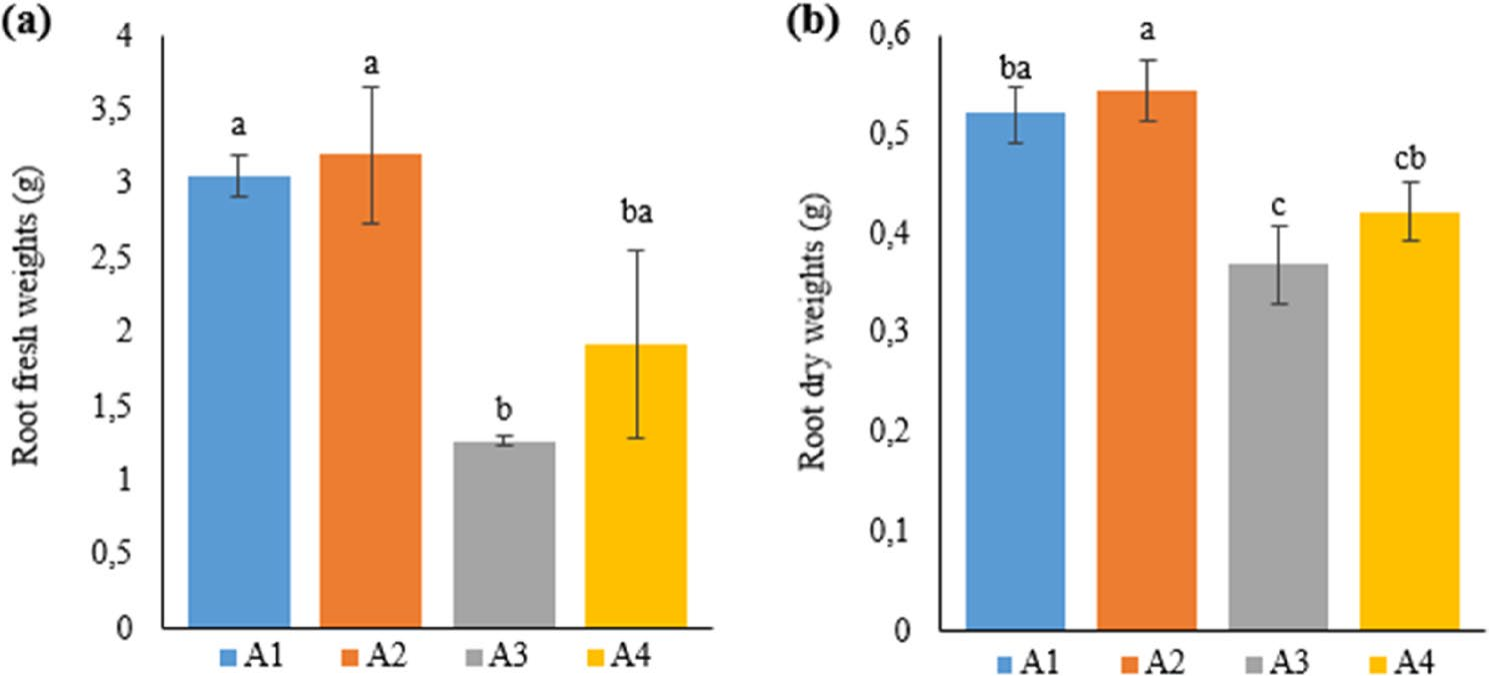
Graph showing the root (**a**) fresh and (**b**) dry weights of cannabis plants which is A1: 1/2 Hoagland + 150:30:150 N: P:K, A2: 1/2 Hoagland + 150:30:150 N: P:K + 10 mg/L Si, A3: 1/2 Hoagland + 150:30:150 N: P:K + 1 mg/L IBA, A4: Soil media (peat: soil: perlite / 1:1:1). Columns shown that mean comparsions were conducted using Duncan’s multiple range test wtih the bars stand for standard deviation (SD). According to the analysis of variance, different letters indicate statistically significant differences at *p* ≤ 0.05

The data on the root dry weights of plants in Table 2 were similar to the data on root fresh weight in terms of order; however, there were generally statistical differences in root dry weights between the treatments. Accordingly, the highest root dry weights were found in A2 (0.54 g), A1 (0.52 g), A4 (0.42 g), and A3 (0.37 g), respectively, similar to the root fresh weight. The comparison of the treatment groups as deep water culture and soil medium demonstrated that deep water culture yielded higher results, except for A3 (IBA), and the differences were statistically significant (Fig. 2).

Reserachers reported that the fresh and dry weights of industrial hemp plants varied under different light intensities and that root fresh weight increased. The current study also revealed that there were significant differences in root fresh and dry weights between industrial hemp plants grown in soil and deep water culture, with higher results obtained in deep water culture [18].

Silicon generally positively affects seed germination and plant height in the hemp plant [19]. Likewise, studies on tomato [20], corn [21] and orange [22] plants have determined that Si treatment increases root fresh and dry weights. Similar to the researchers’ findings, the current study also found that Si treatments increased root fresh and dry weight. Among the treatment groups, the highest root fresh/dry weight (3.2 g/0.54 g) was obtained in the Si-treated group. Silicon ensures the development of denser root systems by promoting cell division and elongation [23, 24]. Additionally, silicon positively affects the uptake and transport of macronutrients used by plants, such as calcium, phosphorus, and potassium [25, 26, 27]. We believe that the high results obtained from the Si treatment in the present study arise from the properties above.

IBA is a plant growth regulator from the auxin group, which is responsible for events, such as cell growth and expansion, cell elongation, tissue development, and root formation promotion. Few studies have been conducted on the use and application of IBA for plant development in soil or soilless cultivation. A study to promote root development in the tropical plant *Vitex diversifolia* reported that plants in the IBA-treated group had lower root fresh weights compared to other treatments [28]. Researchers indicated that IBA treatment was ineffective in improving yield and water and nutrient uptake in the melon plant grown in the soilless medium [29]. In support of the researchers, the present study also found that the lowest root fresh/dry weight in the Narlı hemp variety was obtained from the IBA-treated group. Moreover, another study stated that IBA applied in high concentrations in the production of industrial hemp with cuttings was effective in promoting root development [30].

### Plant stem fresh and dry weight

Table 3 contains data on the stem fresh and dry weights of hemp plants grown in the soil and deep water culture medium.

**Table 3:** Fresh and dry weights of stems of hemp plants with different treatments

Treatment	Stem Fresh Weight (g)	Stem Dry Weight (g)
A1	4.8±0.14^a^	1.38±0.04^a^
A2	4.92±0.05^a^	1.37±0.08^a^
A3	3.35±0.36^b^	0.91±0.07^b^
A4	3.87±0.32^b^	1.07±0.09^ba^

A1: 1/2 Hoagland + 150:30:150 N: P:K

A2: 1/2 Hoagland + 150:30:150 N: P:K + 10 mg/L Si

A3: 1/2 Hoagland + 150:30:150 N: P:K + 1 mg/L IBA

A4: Soil media (peat: soil: perlite / 1:1:1)

* Values in each column marked with different letters indicate statistically significant differences at *p* ≤ 0.05

* The mean was calculated from data obtained from 7 plants

In light of the data in Table 3, the highest value was obtained in A2 (4.92 g) upon examining the impacts of different treatments on plant stem fresh weights in hemp plants grown in the soil and soilless media. In terms of stem fresh weight, A2 was followed by A1 (4.8 g) and A4 (3.87 g), respectively. The lowest data in terms of stem fresh weight were obtained from plants in A3 (3.35 g). No statistically significant difference was identified between A2, where the best stem fresh weight was achieved, and A1. Likewise, there was no statistically significant difference between the stem fresh weights of plants grown in A3 (3.35 g) and A4 (3.87 g). However, statistically significant differences were found upon comparing these treatment groups with A1 and A2.

**Fig. 3:**
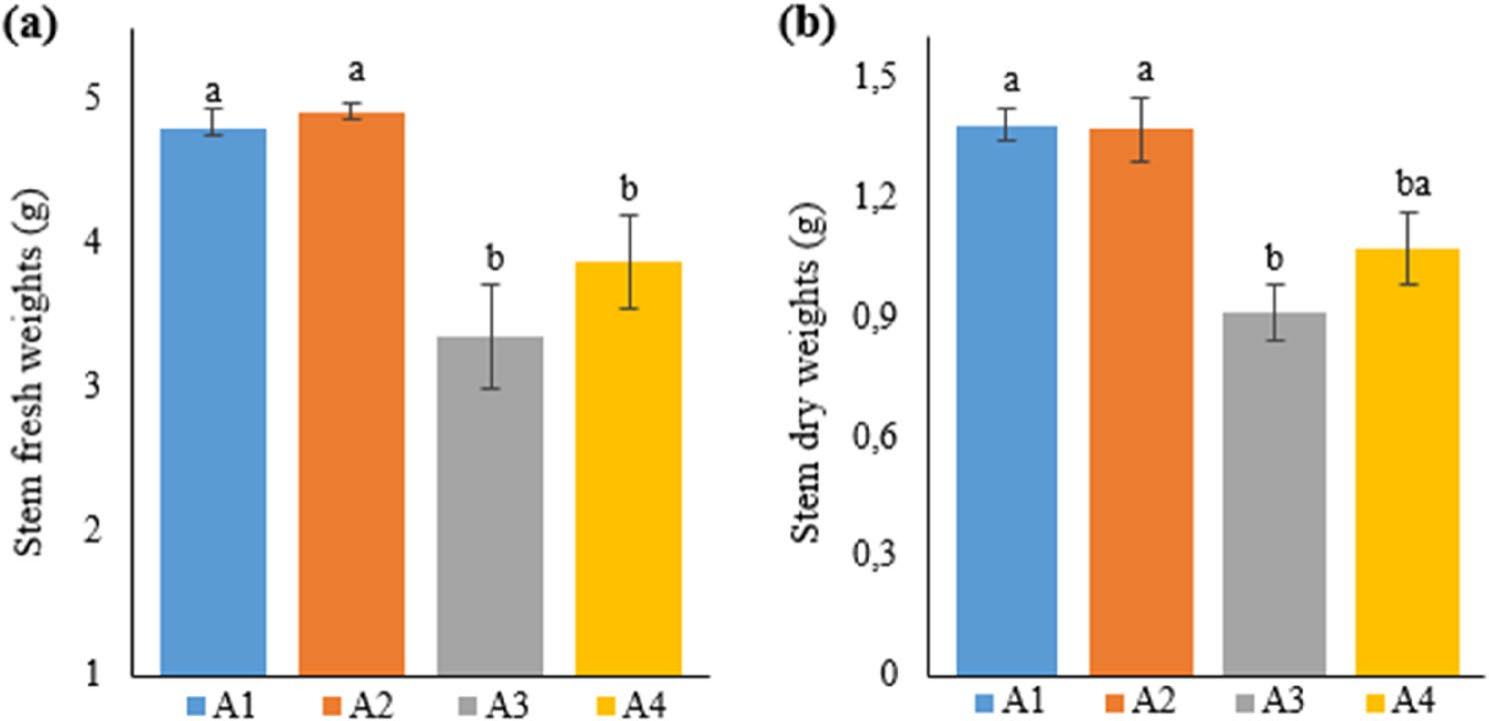
Graph showing the stem (**a**) fresh and (**b**) dry weights of cannabis plants which is A1: 1/2 Hoagland + 150:30:150 N: P:K, A2: 1/2 Hoagland + 150:30:150 N: P:K + 10 mg/L Si, A3: 1/2 Hoagland + 150:30:150 N: P:K + 1 mg/L IBA, A4: Soil media (peat: soil: perlite / 1:1:1). Columns shown that mean comparsions were conducted using Duncan’s multiple range test wtih the bars stand for standard deviation (SD). According to the analysis of variance, different letters indicate statistically significant differences at *p* ≤ 0.05

No significant differences were observed between the treatments in terms of stem dry weight, with the highest to lowest stem dry weight obtained in A1, A2, A4, and A3, respectively. Additionally, the average stem dry weight measured as 0.91 g in IBA-treated (A3) plants decreased statistically significantly in comparison with the other two treatments (A1 and A2). IBA-treated plants, with an average weight of 0.91 g, had the lowest stem dry weight among the groups. The best result with regard to stem dry weight (1.38 g) was obtained from A1 (Fig. 3).

Different LED lights caused changes in the stem fresh and dry weight in industrial hemp [31, 18]. The current study observed differences in the stem fresh and dry weight between the different growing media, hydroponic and soil media.

Stem dry weight increased with increasing N fertilization rates in industrial hemp [32]. Likewise, Campiglia et al. [33] indicated that N treatment in the industrial hemp plant had a positive impact on stem development, increasing biomass. The highest stem dry weight was obtained from hemp plants in Group 1 in the present study, which supports the researchers’ findings.

In a study conducted with the industrial hemp plant in 2021, Luyckx et al. [34] examined changes in plants treated with cadmium and silicon in the hydroponic medium in comparison with the control group. The researchers reported that stem fresh and dry weights decreased in the Si-treated group compared to the control, but this decrease was statistically insignificant. Similar to the researchers’ results, the current study also found that the best result in terms of stem dry weight was obtained in A2 (1.37 g), stem dry weight decreased compared to A1 (1.38 g), but this decrease was statistically insignificant. Furthermore, the best result in stem fresh weight was achieved in A2. Similar to the current study, studies on other plant groups reported that silicon treatments caused increases in stem fresh and dry weights [35, 36].

### Plant stem length

Table 4 presents data on the stem length of Narlı hemp plants grown in the soil and deep water culture medium.

**Table 4:** Plant length of stem of hemp plants with different treatments

Treatment	Plant length (cm)
A1	97.93±4.16^ba^
A2	92.76±3.41^b^
A3	64.64±3.85^c^
A4	113.29±4.43^a^

A1: 1/2 Hoagland + 150:30:150 N: P:K

A2: 1/2 Hoagland + 150:30:150 N: P:K + 10 mg/L Si

A3: 1/2 Hoagland + 150:30:150 N: P:K + 1 mg/L IBA

A4: Soil media (peat: soil: perlite / 1:1:1)

* Values in each column marked with different letters indicate statistically significant differences at *p* ≤ 0.05

* The mean was calculated from data obtained from 7 plants

**Fig. 4:**
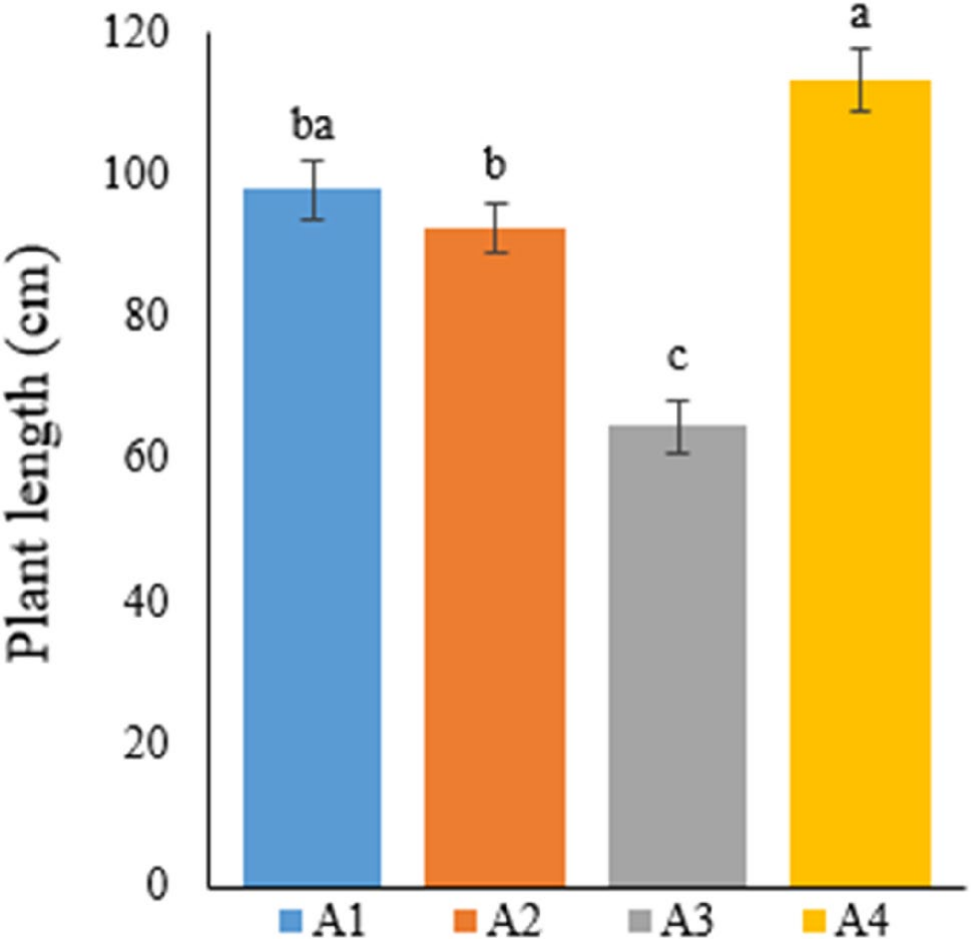
Graph showing the plant length of cannabis plants which is A1: 1/2 Hoagland + 150:30:150 N: P:K, A2: 1/2 Hoagland + 150:30:150 N: P:K + 10 mg/L Si, A3: 1/2 Hoagland + 150:30:150 N: P:K + 1 mg/L IBA, A4: Soil media (peat: soil: perlite / 1:1:1). Columns shown that mean comparsions were conducted using Duncan’s multiple range test wtih the bars stand for standard deviation (SD). According to the analysis of variance, different letters indicate statistically significant differences at *p* ≤ 0.05

Considering the stem length of hemp plants in Table 4, the average stem length of plants grown in the soil medium was measured as 113.29 cm, the highest among all groups. This stem length was followed by A1 (97.93 cm), A2 (92.76 cm), and A3 (64.64 cm), which were hydroponic media (Fig. 4).

Reserchers reported that N treatment in different doses throughout the vegetative growth period in hydroponic media increased stem length in the hemp plant [37]. Likewise, another study stated that nanosilicon treatment increased plant stem length [38]. Si treatment created a difference in plant stem length in the hemp plant grown under hydroponic conditions, but this difference was not statistically significant across all treatment groups [39]. Supporting the researchers’ findings, the highest plant stem length in the current study was obtained from hemp plants grown in soil (A4: 113.29 cm), followed by plants grown in A1 with a stem length of 97.93 cm. The Si element has also been reported to have positive impacts on plant stem length in other plant groups [40, 41].

### Plant stem thickness

Table 5 lists data on the impacts of different growing media on plant stem thickness in hemp.

**Table 5:** Stem thickness of hemp plants with different treatments

Treatment	Stem thickness (cm)
A1	4.96±0.32^ba^
A2	5.54±0.46^a^
A3	4.11±0.15^b^
A4	4.27±0.19^b^

A1: 1/2 Hoagland + 150:30:150 N: P:K

A2: 1/2 Hoagland + 150:30:150 N: P:K + 10 mg/L Si

A3: 1/2 Hoagland + 150:30:150 N: P:K + 1 mg/L IBA

A4: Soil media (peat: soil: perlite / 1:1:1)

*Values in each column marked with different letters indicate statistically significant differences at *p* ≤ 0.05

*The mean was calculated from data obtained from 7 plants

**Fig. 5:**
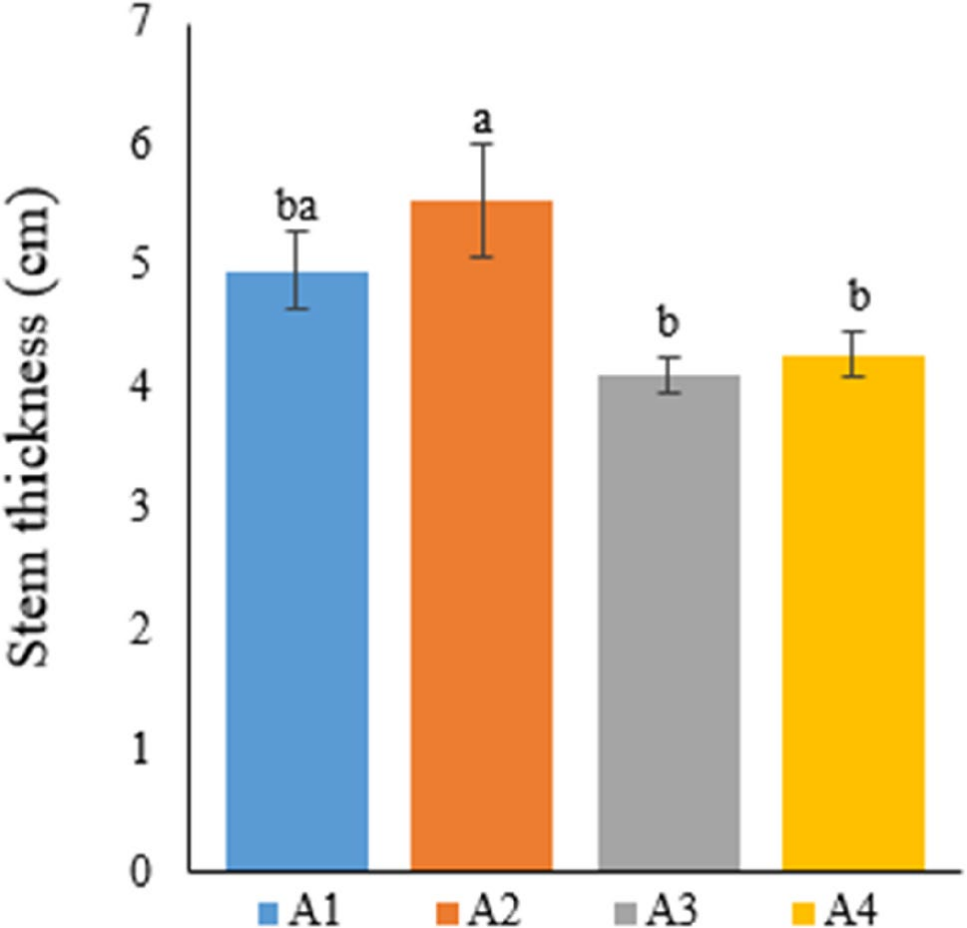
Graph showing the stem thickness of cannabis plants which is A1: 1/2 Hoagland + 150:30:150 N: P:K, A2: 1/2 Hoagland + 150:30:150 N: P:K + 10 mg/L Si, A3: 1/2 Hoagland + 150:30:150 N: P:K + 1 mg/L IBA, A4: Soil media (peat: soil: perlite / 1:1:1). Columns shown that mean comparsions were conducted using Duncan’s multiple range test wtih the bars stand for standard deviation (SD). According to the analysis of variance, different letters indicate statistically significant differences at *p* ≤ 0.05

A review of the data on stem thickness revealed significant differences between hydroponic and soil media. Among all treatment groups, plants with the thickest stems were obtained from A2, with an average stem thickness of 5.54 cm, followed by A1 with an average stem thickness of 4.96 cm. The average stem thickness of plants grown in soil was calculated to be 4.270 cm. The lowest stem thickness (4.11 cm) was obtained from plants in A3. Furthermore, the increase in the average stem thickness of Si-treated plants was found to be statistically significant compared to the soil medium (Fig. 5).

Phantong et al. [42] grew the ornamental plants *Globba schomburgkii* and *Globba marantina* in soil and soilless media for comparison. The researchers reported that the soilless medium was more advantageous than the soil medium in terms of growing conditions. In support of the researchers, the current study also obtained better results in terms of stem thickness in the hydroponic medium in comparison with the soil medium, upon comparing growing conditions (except A3).

Yang et al. [37] indicated that N treatment increased the stem diameter and biomass of the hemp plant. In another study, researchers researched the impacts of Si treatment on Zn stress in the hemp plant grown in the hydroponic medium. Si treatment increased plant stem thickness; however, this increase was found to be insignificant compared to the control and significant compared to Zn stress treatment [43]. The present study found that Si treatment caused an increase in the stem thickness of the hemp plant in the hydroponic medium, and this increase was statistically significant in comparison with the other treatments. Another researcher stated that Si treatment increased stem thickness in the hemp plant [38].

### Plant leaf length

Table 6 demonstrates the impact of the different growing media on the leaf length of the hemp plant in this study.

**Table 6:** Leaf length of hemp plants with different treatments

Treatment	Leaf length (cm)
A1	16.12±1.86^a^
A2	15.4±1.77^a^
A3	10.64±0.30^b^
A4	12.5±0.52^ba^

A1: 1/2 Hoagland + 150:30:150 N: P:K

A2: 1/2 Hoagland + 150:30:150 N: P:K + 10 mg/L Si

A3: 1/2 Hoagland + 150:30:150 N: P:K + 1 mg/L IBA

A4: Soil media (peat: soil: perlite / 1:1:1)

* Values in each column marked with different letters indicate statistically significant differences at *p* ≤ 0.05

* The mean was calculated from data obtained from 3 plants

**Fig. 6:**
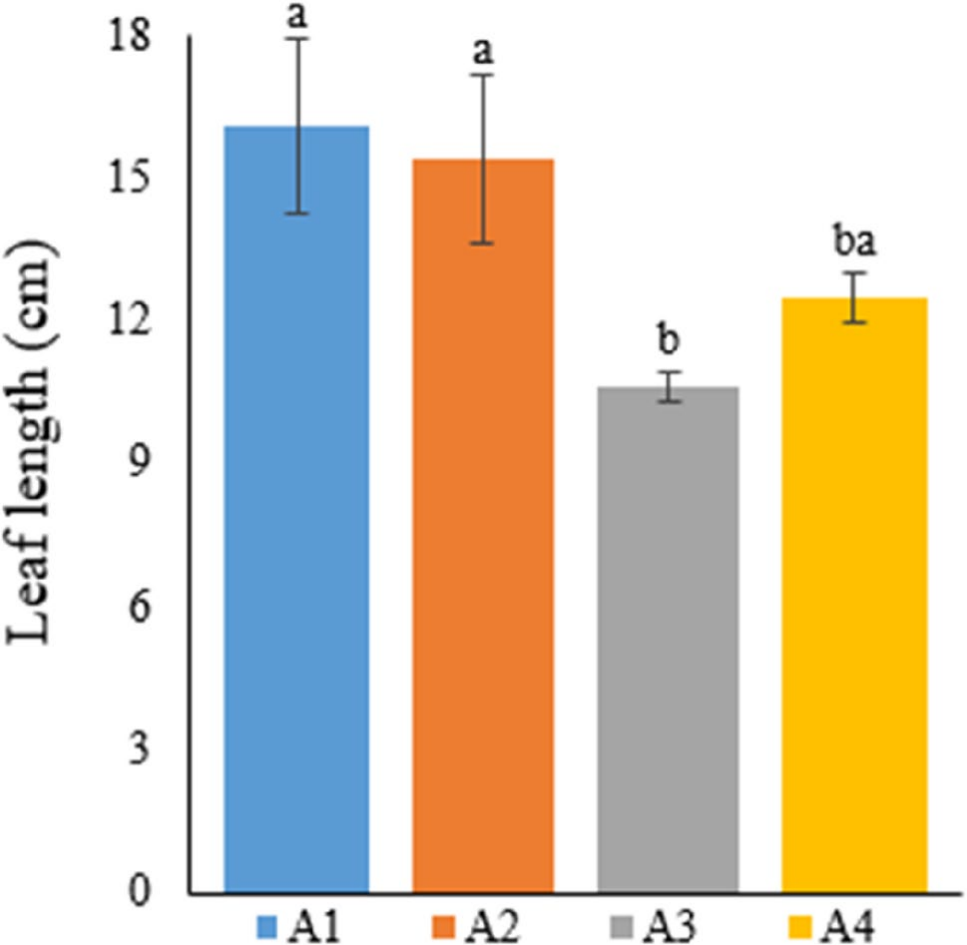
Graph showing the leaf length of cannabis plants which is A1: 1/2 Hoagland + 150:30:150 N: P:K, A2: 1/2 Hoagland + 150:30:150 N: P:K + 10 mg/L Si, A3: 1/2 Hoagland + 150:30:150 N: P:K + 1 mg/L IBA, A4: Soil media (peat: soil: perlite / 1:1:1). Columns shown that mean comparsions were conducted using Duncan’s multiple range test wtih the bars stand for standard deviation (SD). According to the analysis of variance, different letters indicate statistically significant differences at *p* ≤ 0.05

Among all treatments, the highest results in terms of leaf length (16.12 cm) were obtained from plants in A1, followed by plants in A2, A4, and A3, respectively. Leaf length, measured as 10.64 cm in A3, was the lowest value among all treatments (Fig. 6).

In their study, where they grew ornamental plants in soil and soilless media for comparison, Phantong et al. [42] reported that plants grown in the soilless medium had a higher leaf area than those grown in the soil medium. the current study determined that the hydroponic medium yielded better results in terms of both leaf length and width than the soil medium in the hemp plant.

In their study on the rice plant, Babu et al. [44] indicated that Si treatment caused an increase in leaf length. The present study also showed that Si treatment in the hydroponic medium increased the leaf length of the hemp plant in comparison with the soil medium. Additionally, Ashraf et al. [45] reported that the effects of IBA on leaf length and width in radish were statistically insignificant. Similar to the researchers, in the current study found that IBA treatment yielded the lowest results in terms of leaf length and width and had no impact on these parameters.

### Leaf relative water content (%RWC)

Table 7 provides the relative water content (%RWC) in the leaves of Narlı hemp plants grown in the soil and deep water culture medium.

**Table 7:** Relative water content of hemp leaves with different treatments

Treatment	RWC (%)
A1	88.67±1.52^ba^
A2	94.29±2.88^a^
A3	71.54±4.66^c^
A4	80.67±2.60^cb^

A1: 1/2 Hoagland + 150:30:150 N:P:K

A2: 1/2 Hoagland + 150:30:150 N:P:K + 10 mg/L Si

A3: 1/2 Hoagland + 150:30:150 N:P:K + 1 mg/L IBA

A4: Soil media (peat:soil:perlite / 1:1:1)

* Values in each column marked with different letters indicate statistically significant differences at p ≤ 0.05

* The mean was calculated from data obtained from 3 plants

**Fig. 7:**
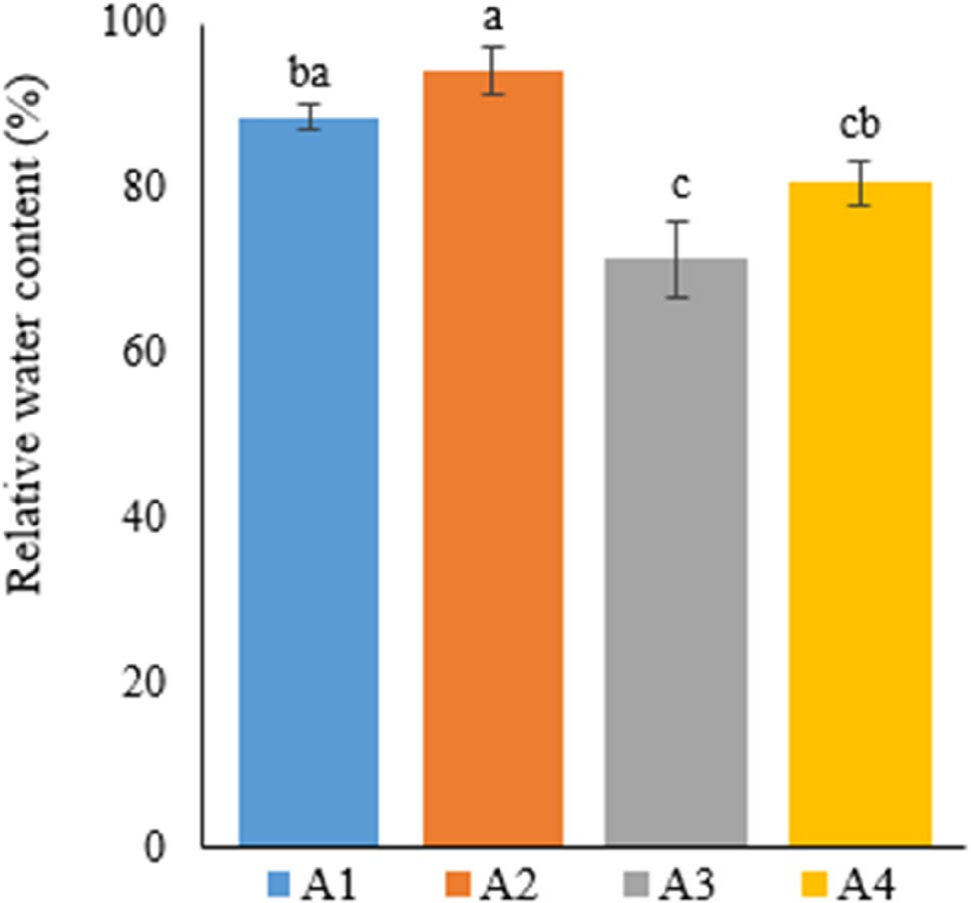
Graph showing the relative water content of cannabis plants which is A1: 1/2 Hoagland + 150:30:150 N: P:K, A2: 1/2 Hoagland + 150:30:150 N: P:K + 10 mg/L Si, A3: 1/2 Hoagland + 150:30:150 N: P:K + 1 mg/L IBA, A4: Soil media (peat: soil: perlite / 1:1:1). Columns shown that mean comparsions were conducted using Duncan’s multiple range test wtih the bars stand for standard deviation (SD). According to the analysis of variance, different letters indicate statistically significant differences at *p* ≤ 0.05

Plants in Group 2 had the highest value in terms of leaf relative water content (94.29%). Subsequently, %RWC was measured as 88.67% in Group 1, 80.67% in Group 4, and 71.54% in Group 3, respectively. The difference between Group 2, with the highest %RWC, and Group 4, the soil medium, was statistically significant. The current study determined that the growing medium was important in terms of %RWC and the hydroponic medium (except Group 3 treated with IBA) was preferable to the soil medium. In practice, the lowest %RWC value was obtained from IBA-treated plants (Fig. 7).

Luyckx et al. [46] researched the impacts of Si treatment on the industrial hemp plant grown under zinc stress in the hydroponic medium. The researchers indicated that water content increased in the Si-treated group in comparison with other treatments, but this increase was statistically insignificant. In the present thesis study, the best %RWC value was obtained from Si treatment, which supports the researchers’ results. Furthermore, silicon treatment created a statistically significant difference compared to the soil medium. Studies conducted on Si in other groups of plants reported that Si treatment increased %RWC in a statistically significant way in comparison with groups not treated with Si [47, 48, 20].

Silicon plays an essential role in many aspects of plant growth and development. Although studies in the literature have generally examined the effects of silicon on plants under stress conditions, they have shown that Si treatment increases leaf %RWC. The current thesis study found that Si treatment increased leaf relative water content in the hemp plant, which agrees with other studies.

### Plant photosynthetic pigment content

Photosynthetic pigment contents in the leaves of Narlı hemp plants grown in the soil and deep water culture media were determined by measuring chlorophyll a, chlorophyll b, and carotenoid values, ​​and are given in Table 8.

**Table 8:** Phtosytnhetic pigment content of hemp leaves with different treatments

Treatment	Chlorophyll-a (mg/g FW)	Chlorophyll-b (mg/g FW)	Total carotenoid (µg/g FW)
Grup-1	1.4±0.02^b^	0.57±0.04^b^	4.52±021^b^
Grup-2	1.5±0.07^a^	0.69±0.04^a^	5.07±063^a^
Grup-3	1.09±0.06^d^	0.46±0.04^b^	3.6±044^d^
Grup-4	1.25±0.02^c^	0.54±0.08^b^	4.14±0.35^c^

A1: 1/2 Hoagland + 150:30:150 N: P:K

A2: 1/2 Hoagland + 150:30:150 N: P:K + 10 mg/L Si

A3: 1/2 Hoagland + 150:30:150 N: P:K + 1 mg/L IBA

A4: Soil media (peat: soil: perlite / 1:1:1)

* Values in each column marked with different letters indicate statistically significant differences at *p* ≤ 0.05

* Fresh leaf samples (250 mg) were obtained from 7 plants and analyzed 3 replicates (*n* = 7)

Considering the pigment content of hemp plants grown in the soil and soilless media, the highest chlorophyll a content was obtained in A2 (1.5 mg/g FW), followed by A1 (1.4 mg/g FW), A4 (1.25 mg/g FW), and A3 (1.09 mg/g FW), respectively. These differences in chlorophyll a content between each treatment were statistically significant. Moreover, the difference in chlorophyll a content in A2 was revealed to be statistically significant compared to all other groups.

Similar to chlorophyll a content, chlorophyll b content was ranked from highest to lowest as A2, A1, A4, and A3 (0.69 mg/g FW, 0.57 mg/g FW, 0.54 mg/g FW, and 0.46 mg/g FW, respectively). Unlike the cholorophyll a content, differences between A1, A3, and A4 in terms of chlorophyll b content were statistically insignificant. Furthermore, A2, where the best data were obtained, differed from the other three groups, and this difference was statistically significant.

**Fig. 8:**
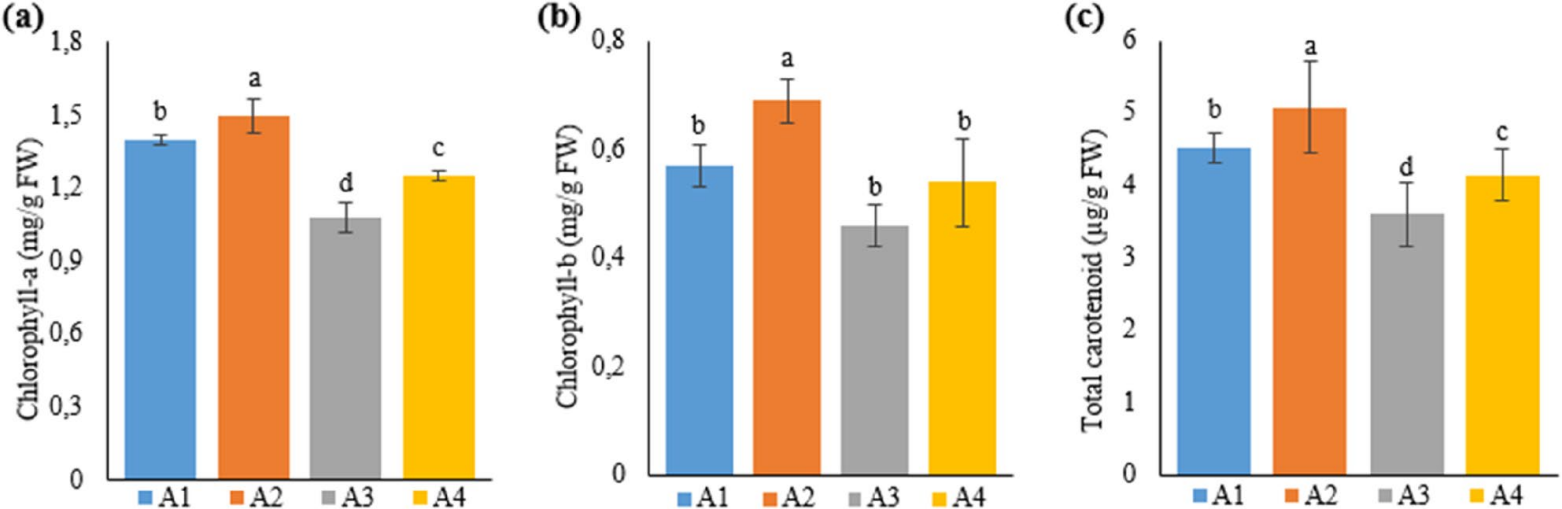
Graph showing the chlorophyll-a (**a**), chlorophyll-b (**b**) and total carotenoid (**c**) content of cannabis plants which is A1: 1/2 Hoagland + 150:30:150 N: P:K, A2: 1/2 Hoagland + 150:30:150 N: P:K + 10 mg/L Si, A3: 1/2 Hoagland + 150:30:150 N: P:K + 1 mg/L IBA, A4: Soil media (peat: soil: perlite / 1:1:1). Columns shown that mean comparsions were conducted using Duncan’s multiple range test wtih the bars stand for standard deviation (SD). According to the analysis of variance, different letters indicate statistically significant differences at *p* ≤ 0.05

Concerning total carotenoid content, values were ranked from highest to lowest as A2, A1, A4, and A3 (5.07 µg/g FW, 4.52 µg/g FW, 4.14 µg/g FW, and 3.6 µg/g FW, respectively). As chlorophyll a content, total carotenoid content also differed among the groups, and the differences between the groups were statistically significant. The highest carotenoid content was obtained from A2, similar to chlorophyll a and b (Fig. 8).

Researchers investigated the impacts of the Si element on the growth and development parameters in hemp plants grown in different growing media [49]. The researchers reported that physiological and morphological parameters increased in Si-treated plants compared to untreated ones, and the hemp plant grown in the soilless growing medium was more productive than the plant grown in the soil medium. Supporting the researchers’ findings, the current study determined that Si-treated hemp plants grown in the soilless medium had the highest values in terms of pigment content. Another study carried out in a soilless medium found that the increased Si concentration (2.5 mg/L, 5 mg/L) increased chlorophyll a, chlorophyll b, and carotenoid contents in *Camelina sativa* L [39].

Likewise, in their study on the impacts of drought stress on the mango plant, Helaly et al. [46] reported that Si increased pigment content in comparison with the control and plants under stress. Xavier et al. [50] stated that total chlorophyll content increased in cucumber (*Cucumis sativus* L.) treated with different doses of silicon compared to controls, depending on the dose applied. Similar to the studies above, the current study also found that Si treatment increased pigment content.

Si displays its positive effects on plant growth and development by increasing pigment content. In line with studies in the literature, the present study also revealed that Si treatment increased leaf pigment (chlorophyll a, chlorophyll b, and carotenoid) contents.

### Determination of leaf lipid peroxidation on leaves (MDA content)

MDA content was studied in the Narlı industrial hemp plant grown in the soil and deep water culture media to determine the effects of growing conditions and different treatments on plant development and the degree of cell damage (Table 9).

**Table 9:** MDA content of hemp leaves with different treatments

Treatment	MDA content (µmol/g FW)
A1	2.72±0.12^c^
A2	2.81±0.08^c^
A3	6.45±0.28^a^
A4	4.28±0.16^b^

A1: 1/2 Hoagland + 150:30:150 N: P:K

A2: 1/2 Hoagland + 150:30:150 N: P:K + 10 mg/L Si

A3: 1/2 Hoagland + 150:30:150 N: P:K + 1 mg/L IBA

A4: Soil media (peat: soil: perlite / 1:1:1)

* Values in each column marked with different letters indicate statistically significant differences at *p* ≤ 0.05

* Fresh leaf samples (100 mg) were obtained from 7 plants and analyzed 3 replicates (*n* = 7)

When the treatments were compared, the highest MDA content was measured in hemp plants in A3 as 6.45 µmol/g. This value showed that IBA treatment caused the most cell membrane destruction in the plant in comparison with the other treatments. The MDA content in hemp plants grown in soil was measured as 4.28 µmol/g, making it the treatment with the highest cell membrane destruction after IBA.

**Fig. 9:**
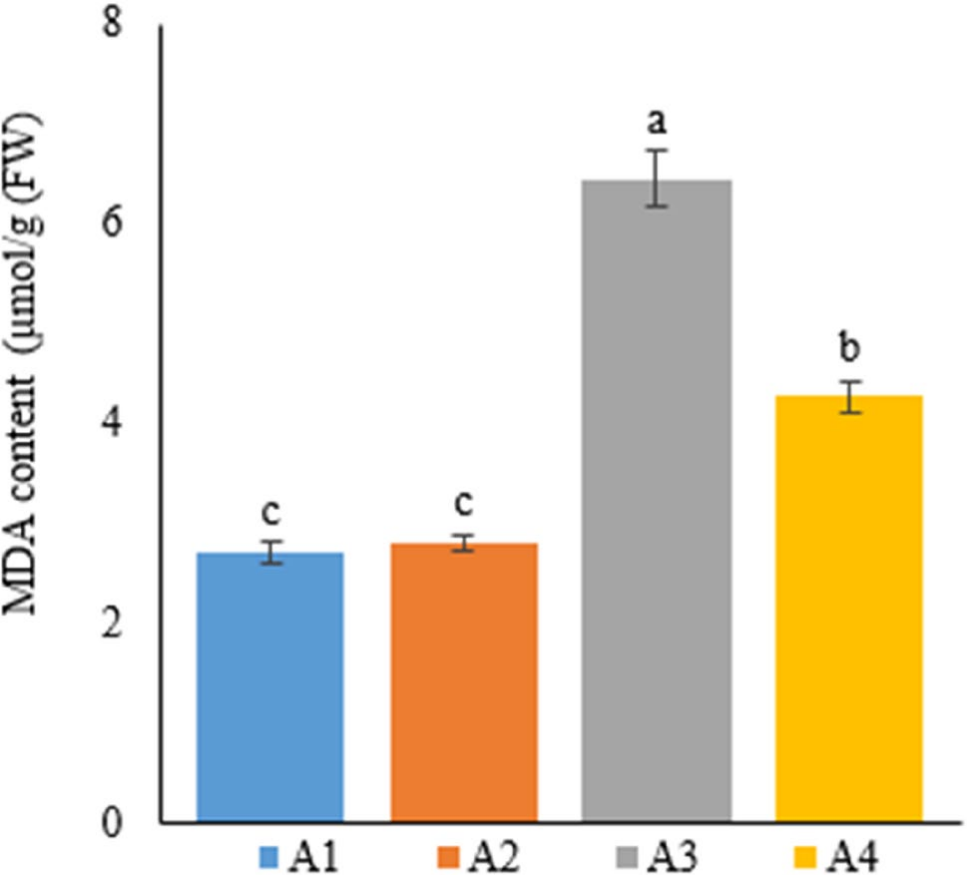
Graph showing the malondialdehyde content of cannabis plants which is A1: 1/2 Hoagland + 150:30:150 N: P:K, A2: 1/2 Hoagland + 150:30:150 N: P:K + 10 mg/L Si, A3: 1/2 Hoagland + 150:30:150 N: P:K + 1 mg/L IBA, A4: Soil media (peat: soil: perlite / 1:1:1). Columns shown that mean comparsions were conducted using Duncan’s multiple range test wtih the bars stand for standard deviation (SD). According to the analysis of variance, different letters indicate statistically significant differences at *p* ≤ 0.05

In hydroponically grown plants in A1 and A2, MDA content was about three times lower than that in A3 and about two times lower than that in A4. These differences between A1 and A2 and between A3 and A4 were statistically significant, but the difference between the two treatments with the lowest MDA content (A1 and A2) was statistically insignificant (Fig. 9).

MDA is a product of cell membrane destruction and an essential stress parameter in plants. In a study on industrial hemp, Luyckx et al. [46] treated plants grown in deep water culture with Zn and Si. Accordingly, they reported that Si treatment reduced MDA content in the plant compared to the control group, not treated with Zn or Si. In a similar study, Luyckx et al. [34] treated the industrial hemp plant grown in the hydroponic medium with Cd and investigated the impacts of Si treatment on the plant. The researchers indicated that Si treatment, compared to Cd treatment, reduced MDA content. The current study also measured the lowest content of MDA, the end product of cell membrane destruction in the industrial hemp plant, in plants in A1 and A2 grown in deep water culture, but did not find the difference between these two groups to be statistically significant. Furthermore, in support of the current findings, similar results have been reported in other plant groups, and Si treatment was stated to reduce MDA content [22, 51].

Studies in the literature have generally investigated the effects of the Si element on stress parameters, indicating results parallel to the current thesis study. According to these results, Si treatment reduced cell membrane damage in the plant, and the MDA content was significantly lower in the plant leaves in the Si-treated growing medium compared to the other groups. Additionally, IBA treatment caused cell membrane damage in the plant in comparison with other treatments.

### Leaf secondary metabolites (CBD, Δ^9^THC, and CBN) content

Leaf secondary metabolite contents were studied in the Narlı variety of the industrial hemp (*Cannabis sativa* L.) plant to investigate what changes different growing media (soil and hydroponics) and different nutrient contents in the growing media caused in the content of some secondary metabolites (CBD, Δ^9^THC, and CBN) in the plant. The results are presented in Table 10.

**Table 10:** CBD, Δ^9^THC, CBN content of hemp leaves with different treatments

Treatment	CBD (%w/w)	Δ^9^THC (%w/w)	CBN (%w/w)
A1	0.0213±0.002^b^	0.002±0.0003^b^	0.0003±0.00003^b^
A2	0.0134±0.001^b^	0.0017±0.0003^b^	0.0002±0.00003^b^
A3	0.0395±0.004^a^	0.0032±0.0006^a^	0.0002±0.00003^b^
A4	0.0326±0.003^a^	0.0033±0.0007^a^	0.0009±0.00012^a^

A1: 1/2 Hoagland + 150:30:150 N:P:K

A2: 1/2 Hoagland + 150:30:150 N:P:K + 10 mg/L Si

A3: 1/2 Hoagland + 150:30:150 N:P:K + 1 mg/L IBA

A4: Soil media (peat:soil:perlite / 1:1:1)

* Values in each column marked with different letters indicate statistically significant differences at p ≤ 0.05

* Dry leaf samples (3 g) were obtained from 7 plants (n = 7)

**Fig. 10:**
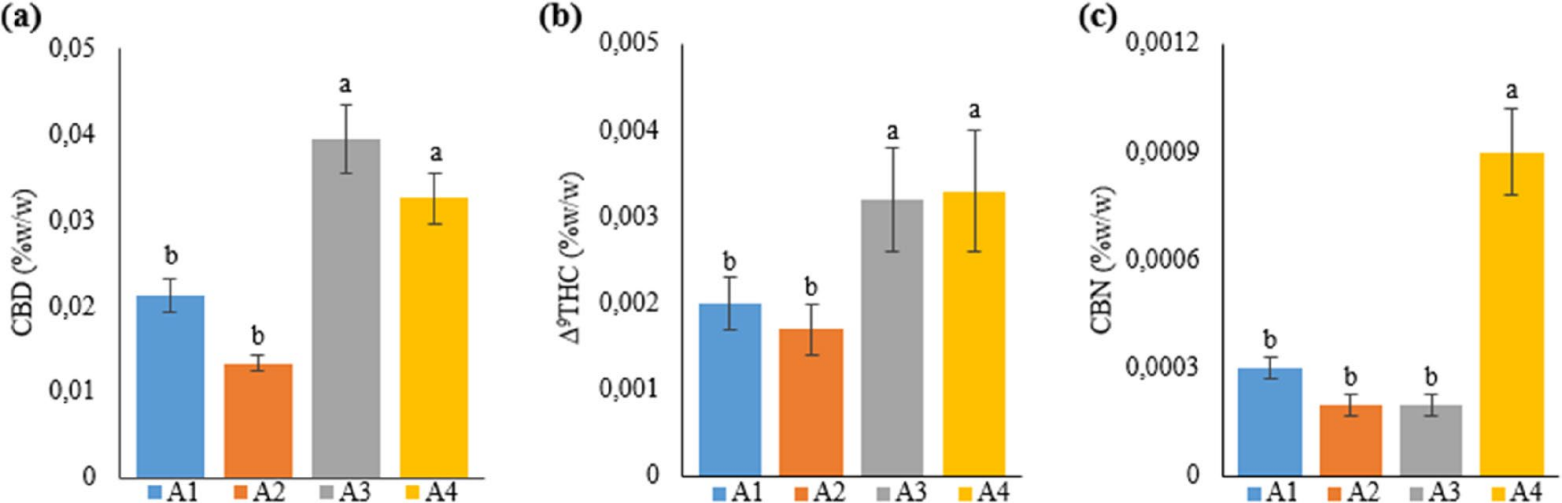
Graph showing CBD (**a**), Δ^9^THC (**b**) and CBN (**c**) content of cannabis plants which is A1: 1/2 Hoagland + 150:30:150 N: P:K, A2: 1/2 Hoagland + 150:30:150 N: P:K + 10 mg/L Si, A3: 1/2 Hoagland + 150:30:150 N: P:K + 1 mg/L IBA, A4: Soil media (peat: soil: perlite / 1:1:1). Columns shown that mean comparsions were conducted using Duncan’s multiple range test wtih the bars stand for standard deviation (SD). According to the analysis of variance, different letters indicate statistically significant differences at *p* ≤ 0.05

Upon examining the secondary metabolite content in terms of CBD, the results were ranked from highest to lowest as A3, A4, A1, and A2, respectively. Groups A3 and A4 had the highest CBD content, and there was no statistically significant difference between these groups. Additionally, a statistically significant difference was found between A1 and A2 and between A3 and A4 in terms of CBD content; however, no significant difference was observed between A1 and A2 (Fig. 10).

As seen in Table 10, the Δ^9^THC content between the groups is parallel to the CBD content. The Δ^9^THC content in A3 and A4 was about 1.5-2 times higher than that in A1 and A2 treatments. Likewise, whereas no statistically significant difference was found between A4 and A3 and between A1 and A2, the difference in Δ^9^THC content was significant upon comparing A3 and A4 with A1 and A2 (Fig. 10).

The highest CBN content (unlike the Δ^9^THC and CBD content) was obtained from the hemp plant in A4, as 0.0009%. The difference between the CBN content in A4 and the CBN content of plants in the other three groups was found to be statistically significant, and the contents were ranked from highest to lowest as 0.0003%, 0.0002%, and 0.0002% in A1, A2, and A3, respectively. Additionally, no significant difference was found between CBN contents in plants in A1, A2, and A3 (Fig. 10).

The industrial classification of the hemp plant is based on THC content in percentage, and the lowest THC threshold value at which no psychoactive impacts are observed is accepted to be 1% [52]. In line with this information, the THC values of the local Narlı variety of industrial hemp used in the current thesis study were found to be quite low, below 1%, in all treated groups.

Researchers studied the impacts of different Si substrate additions on micronutrient uptake, plant growth, and cannabinoid content in hemp plants grown in a greenhouse. They reported that calcium silicate (CaSiO_3_) added to the plant growth medium in different concentrations caused a decrease in total THC content, but this decrease was statistically insignificant [53]. Another study [19] investigated the effects of silicon nanoparticle treatment on four different hemp varieties. The researcher cultivated the industrial hemp plant in two different substrates and reported that Si treatment reduced THC and CBD contents in some groups, while having no impact on others. In another similar study, Rezghiyan et al. [38] investigated the effects of nano-silicon treatment on terpenoid and CBD contents in the hemp plant under drought stress. The researchers stated that CBD content decreased in Si-treated groups in comparison with untreated groups, and this decrease was statistically significant depending on the degree of stress. The present thesis study obtained similar results, supporting the findings of the studies above, and found that Si treatment generally reduced CBD, Δ^9^THC, and CBN contents. Furthermore, Trajkovska et al. [54] reported that different concentrations of IBA caused a decrease in CBD and THC contents in the hemp plant grown in the in vitro culture medium. Contrary to the researchers’ findings, in the current study found that IBA treatment acted as a stress factor in the hemp plant, causing an increase in CBD, Δ^9^THC, and CBN contents.

The present thesis study generally parallels the limited number of studies in the literature on the impacts of Si treatment on cannabinoid content in the hemp plant. Considering that silicon treatment reduces the adverse effects of stress on plants and increases plant development, this thesis study showed that Si treatment caused a reduction in the content of secondary metabolites, increasing under stress.

### Fiber yield

Fiber yield was determined in light of the data on the fiber content and cellulose content of the hemp plant grown in the soil and deep water culture media.

#### Fiber content (%w/w)

Table 11 shows fiber content, one of the most important parameters in the Narlı variety of the industrial hemp plant, in percentage by weight.

**Table 11:** Fiber content of hemp stems with different treatments

Treatment	Fiber content (%w/w)
A1	24.02±2.77^ba^
A2	29.12±3.36^a^
A3	13.74±1.58^c^
A4	19.98±2.30^cb^

A1: 1/2 Hoagland + 150:30:150 N: P:K

A2: 1/2 Hoagland + 150:30:150 N: P:K + 10 mg/L Si

A3: 1/2 Hoagland + 150:30:150 N: P:K + 1 mg/L IBA

A4: Soil media (peat: soil: perlite / 1:1:1)

* Values in each column marked with different letters indicate statistically significant differences at *p* ≤ 0.05

* The mean was calculated from data obtained from 7 plants

**Fig. 11:**
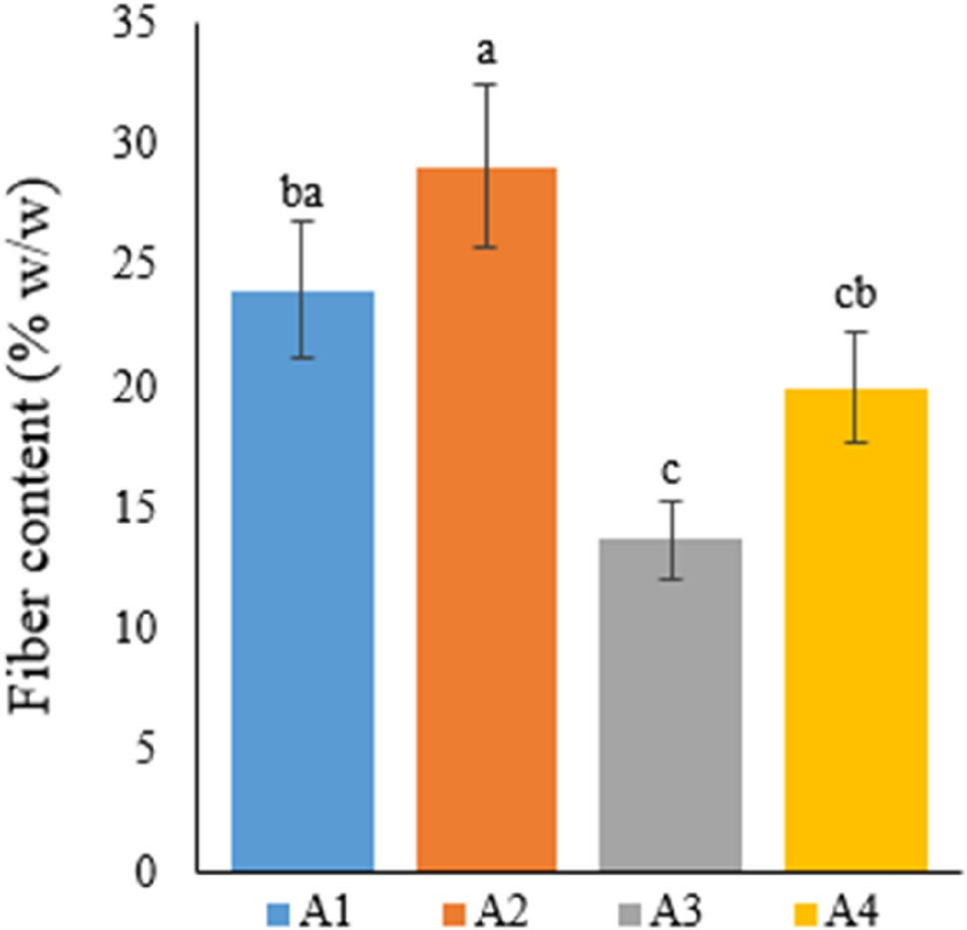
Graph showing the fiber content of cannabis plants which is A1: 1/2 Hoagland + 150:30:150 N: P:K, A2: 1/2 Hoagland + 150:30:150 N: P:K + 10 mg/L Si, A3: 1/2 Hoagland + 150:30:150 N: P:K + 1 mg/L IBA, A4: Soil media (peat: soil: perlite / 1:1:1). Columns shown that mean comparsions were conducted using Duncan’s multiple range test wtih the bars stand for standard deviation (SD). According to the analysis of variance, different letters indicate statistically significant differences at *p* ≤ 0.05

According to these results, the highest fiber content (29.12%) was obtained from the hemp plant in A2. The fiber content in A2 was followed by a fiber content of 24.02% in A1 and a fiber content of 19.98% in A4. A3 was the treatment with the lowest fiber content of 13.73%. This study, conducted using two different growing media, soil and deep water culture, found that A2 containing Si was the most productive medium in terms of fiber content, and the difference between A3 and A4 was statistically significant. Moreover, after the Si treatment, the most efficient result in terms of fiber yield was obtained from A1, and there was no statistical difference between these two groups. The %fiber content obtained from the Si treatment was approximately 2.5 times higher than the fiber content of the IBA-treated hemp and approximately 1.5 times higher than the fiber content of the hemp plants grown in soil (Fig. 11).

Diverse methods are employed to separate fibers from hemp plants, such as soaking in dew, mechanical separation, chemical treatment, retting, and enzyme treatment [55]. Among the methods above, the traditional retting method yields the most successful results in fiber yield. In their study investigating the impacts of fiber separation methods on some quality parameters in hemp fibers, Jankauskiene et al. [56] indicated the positive impact of the water retting method on fiber quality. The current study also used the water retting method, ensuring that the hemp stem, separated from its leaves, was placed in pools of fresh, clean water to separate the woody parts from fibers. Fibers were separated from the plant’s woody parts at 100% by keeping the hemp plant in water at 25–30 °C for 3–5 days.

Researchers cultivated hemp to examine the differences between various hemp varieties in terms of fiber and seed yield. At the end of a four-month growing period, the researchers reported the highest fiber contents as 27.3% in the Santhica 27 variety, 26.5% in the Tygra variety, and 27.1% in the Bialobrzeskie variety [15]. In the current study, approximately 29% fiber content was obtained in the Narlı hemp variety from the Si-treated deep water culture medium. The high fiber content obtained when compared with the literature constitutes significant data. In another study, Deleuran and Frengmark [57] stated that the lowest fiber content was 22.1% and the highest was 33.8% in two different regions of Denmark, with harvest times varying between 4 and 5 months, depending on the variety and conditions. In the present study, 24% (A1) and 29% (A2) fiber contents were obtained from the Narlı hemp variety in the deep water culture medium for approximately 2 months. In comparison to studies in the literature, the current research’s achievement of a higher fiber content in a shorter period is a remarkable result.

Luyckx et al. [43] investigated the effects of silicon treatment on industrial hemp plants in soil containing and not containing heavy metals and reported that Si treatment did not statistically impact the %fiber content of the plant. Contrary to the researchers’ findings, in the current study, the highest fiber content was obtained from A2 treated with Si, and the difference in fiber percentage between plants grown in the Si-treated deep water culture and soil was statistically significant.

When studies are evaluated as a whole and compared with the literature, it can be concluded that the %fiber content, which is crucial for industrial hemp plants, varies depending on the variety and growing conditions.

#### Total cellulose content (%w/w)

Table 12 presents total cellulose content, which is one of the parameters determining fiber quality, of the Narlı variety of industrial hemp plant grown in soil and deep water culture, by weight.

**Table 12:** Total cellulose content of hemp fibers with different treatments

Treatment	Total Cellulose Content % (w/w)
A1	77.25±3.41^ba^
A2	82.49±2.89^a^
A3	61.59±2.97^b^
A4	73.85±3.78^ba^

A1: 1/2 Hoagland + 150:30:150 N:P:K

A2: 1/2 Hoagland + 150:30:150 N:P:K + 10 mg/L Si

A3: 1/2 Hoagland + 150:30:150 N:P:K + 1 mg/L IBA

A4: Soil media (peat:soil:perlite / 1:1:1)

* Values in each column marked with different letters indicate statistically significant differences at p ≤ 0.05

* The mean was calculated from data obtained from 7 plants

**Fig. 12:**
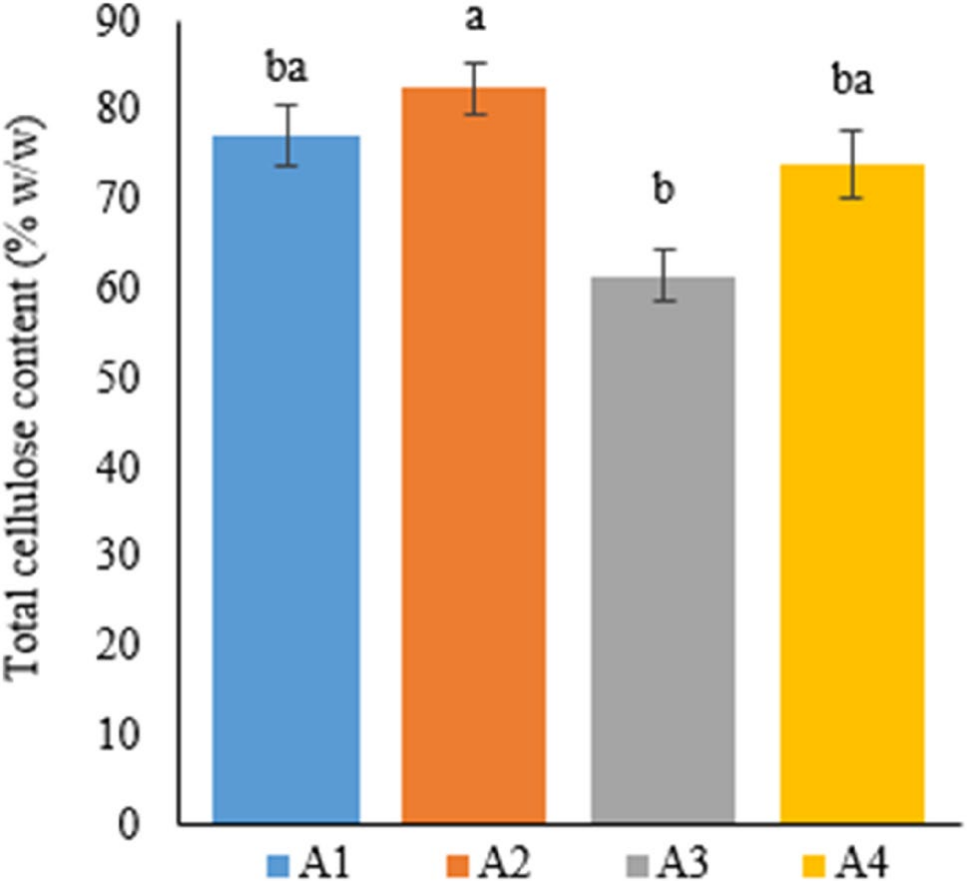
Graph showing the total cellulose content of cannabis plants which is A1: 1/2 Hoagland + 150:30:150 N: P:K, A2: 1/2 Hoagland + 150:30:150 N: P:K + 10 mg/L Si, A3: 1/2 Hoagland + 150:30:150 N: P:K + 1 mg/L IBA, A4: Soil media (peat: soil: perlite / 1:1:1). Columns shown that mean comparsions were conducted using Duncan’s multiple range test wtih the bars stand for standard deviation (SD). According to the analysis of variance, different letters indicate statistically significant differences at *p* ≤ 0.05

An analysis of the data in Table 12 shows that cellulose content varies by treatment, with A2 having the highest total cellulose content of 82.49%. The total cellulose content in A2 was followed by A1, A4, and A3 (77.25%, 73.85%, and 61.59%, respectively). The total cellulose content in Si-treated plants was computed to be approximately 34% higher than that of IBA-treated plants, which had the lowest total cellulose content, and this difference was statistically significant (Fig. 12).

Cellulose, which is the main component of hemp fibers, has essential properties such as fiber absorbency, dyeability, chemical modification capability, and suitability for fiber processing [58]. To this end, the present study examined total cellulose content, a parameter determining fiber quality. Studies conducted on hemp in Türkiye have not used hydroponic media, which makes the results of the current research particularly important.

In their study, Manaia et al. [59] reported that cellulose content was 70–85% in hemp (*Cannabis sativa* L.). Jaldon et al. [60] examined the total cellulose content of fibers obtained from hemp stems. Accordingly, the researchers reported a total cellulose content of 71%. Jankauskienė et al. [56] indicated the total cellulose content in hemp fibers separated using two different pooling procedures to be in the range of 84–88%. Among similar studies, Thomson et al. [61] reported the total cellulose content in hemp fibers as 80% to 86%, and Smole et al. [62] as 77%.

According to data in the literature, the cellulose content in the fibers of different hemp varieties ranges from a minimum of 70% to a maximum of 86%. In the current thesis, in line with the literature, the total cellulose content in the fibers obtained from the stems of hemp plants grown under different treatments varied between 61% and 82% on average. It is also a remarkable result that a total cellulose content of 82% was obtained from plants grown in the Si-treated deep water culture medium at the end of a two-month vegetation period.

## Conclusion

Hemp, which is a valuable plant with superiorities in almost all areas of use, has fibers of the highest quality. This study, carried out to increase the plant’s yield and quality, aimed to improve fiber yield using soilless agriculture (deep water culture). The soilless medium yielded better results than the soil medium in all the parameters tested. However, IBA treatment adversely affected plant development and fiber yield in the soilless medium. Among the treatments in the soilless medium, the hemp plants grown in the deep water culture containing Si yielded better results in terms of both fiber yield (29% fiber content) and fiber quality (82% total cellulose content) than those grown in the soil medium.

Moreover, considering the vegetation period of the hemp plant, seeds were sown in the soil and soilless media after a 14-day germination period and harvested after a 62-day development period. In traditional agriculture, compared to the development period of approximately four months, the method employed in the current study offers a considerable advantage in shortening the vegetation period to 2.5 months.

Alternative methods to traditional agriculture should be developed, considering environmental problems such as global warming, drought, and water scarcity. Soilless agriculture is a significant alternative due to its sustainability, yield, and contribution to the environment. The current study presented an applicable alternative using the deep water culture in the cultivation of industrial hemp. Future research should focus on improving total biomass and fiber yield by using different light supply, growing mediums and ambient conditions in soilles agriculture.

## Data Availability

Data is provided within the manuscript.
